# Using the implementation research logic model to examine high-intensity resistance rehabilitation implementation in skilled nursing facilities: a mixed methods multi-site case study

**DOI:** 10.1186/s43058-025-00747-4

**Published:** 2025-05-21

**Authors:** Lauren A. Hinrichs-Kinney, Danielle Derlein, Mattie E. Pontiff, Daniel Malone, Jodi Summers Holtrop, Jennifer E. Stevens-Lapsley

**Affiliations:** 1 Physical Therapy Program, Department of Physical Medicine and Rehabilitation, University of Colorado Anschutz Medical Campus, Aurora, CO USA; 2 VA Eastern Colorado Geriatric Research Education and Clinical Center, Aurora, CO USA; 3 Denver-Seattle Center of Innovation for Veteran Centered and Value Driven Care (COIN), Aurora, CO USA; 4 Department of Family Medicine, University of Colorado, Aurora, CO USA; 5 Adult and Child Center for Outcomes Research and Delivery (ACCORDS), University of Colorado, Aurora, CO USA

**Keywords:** Geriatrics, Skilled nursing facilities, Implementation, Exercise therapy, Rehabilitation

## Abstract

**Background:**

Implementing evidence-based rehabilitation in skilled nursing facilities (SNFs) is essential for enhancing physical function outcomes and mitigating risk of adverse events. Best implementation approaches in this complex setting are unknown. This study uses the Implementation Research Logic Model (IRLM) to retrospectively examine the implementation of high-intensity resistance rehabilitation (HIR) in SNFs, aiming to elucidate contextual factors and pathways that could enhance future HIR implementation endeavors.

**Methods:**

We conducted a convergent, mixed-methods multi-site case study (*n* = 8 sites). A standardized implementation strategy was employed, allowing sites to adapt this approach. HIR use was measured using the Provider Report of Sustainment Scale (PRESS). Contextual factors were identified using the Practical Robust Implementation and Sustainability Model (PRISM) through study-specific questionnaires and validated measures (Inner Setting Scale, Provider Perspective of Team Effectiveness, Evidence Based Practice Attitudes Scale, Perceived Characteristics of Intervention Scale, Self-Defined Burnout Measure, and Utrecht Engagement Scale), and analyzed descriptively. Interviews and focus groups with leadership and clinicians revealed contextual factors and strategies influencing implementation. Heat maps visualized site patterns, while an IRLM proposed provisional implementation pathways.

**Results:**

PRESS scores ranged from 3.75 (0.17) to 2.33 (0.67), indicating all sites implemented HIR to at least a “moderate extent”. Higher-implementing sites demonstrated full-team ability to adapt HIR to diverse patients. Differentiating contextual factors between higher and lower implementing sites included clinician perspectives, site infrastructure, and satisfaction with leadership. Higher-implementing sites employed a higher volume of site-initiated implementation strategies, notably having a champion and patient engagement. Pathways that appeared to contribute to higher implementation extent included: 1) overcoming inertia of current practice through HIR salience, 2) overcoming clinician concerns of patient compatibility through affirmative experiences, 3) addressing clinician perspective of complexity with session planning, and 4) optimizing patient rehabilitation mindset through encouraging environments.

**Conclusion:**

Improving physical function in older adults necessitates adoption of evidence-based rehabilitation like HIR. Implementation strategies that target infrastructure, including leadership support and communication channels, inertia of current practice, and clinician perspectives of HIR complexity and patient compatibility may facilitate implementation. Identifying a champion and providing guidance for effective patient engagement appear to be key.

**Supplementary Information:**

The online version contains supplementary material available at 10.1186/s43058-025-00747-4.

Contributions to the literature • Knowledge on implementing evidence-based programs shown to improve physical function and reduce the risk of adverse events among frail, older adults within the complex and underrepresented context of skilled nursing facility rehabilitation is limited.

• This study identifies the importance of considering strategies that address clinician perspectives, site infrastructure (including leadership support and communication channels), existing practice inertia, and patients’ rehabilitation mindset. These strategies are suggested to promote environmental adaptations and cues, clinician creativity and self-efficacy, continuity of care, and supportive patient environments.

• Findings can inform future efforts aimed at implementing evidence-based rehabilitation practices in skilled nursing facilities.

## Background

The objective of rehabilitation in Skilled Nursing Facilities (SNFs) is to improve physical function for patients who experience hospital-associated functional deficits. Over 60% of patients discharged from SNFs are at functional levels that put them at heightened risk for adverse events [1–4]. Fortunately, physical function is a modifiable risk factor [5, 6], but implementation of rehabilitation interventions that more effectively improve physical function are necessary.

One such rehabilitation intervention is progressive, high-intensity resistance rehabilitation (HIR), which directly targets muscle weakness to improve physical function among patients with medical complexities [7]. HIR involves physiologically overloading skeletal muscle through low-repetition, high-resistance activities. It is safe and effective in improving physical function among older adults with medical complexities, specifically in SNFs [8]. However, its implementation across a limited number of SNFs (*n* = 3) has shown variable success – one site achieved high levels of reach, implementation, and effectiveness, while others demonstrated lower levels across these outcomes [8, 9]. Understanding the key strategies and contextual factors influencing its implementation remains limited [9].

Skilled nursing facilities are underrepresented in implementation research and pose unique challenges given complexity at multiple levels. This includes patients’ medical complexity and variability in functional and cognitive levels; external pressures including the recent shift in reimbursement models and documentation demands; and facility-level issues like staffing shortages, high staff turnover, limited resources, poor organizational culture, and care models where patients are treated by multiple rehabilitation professionals within the same discipline [9–13]. Such challenges paired with the variability across SNFs [14] makes examination of HIR implementation at the facility or site level essential requiring consideration of team dynamics and the physical and sociocultural environmental context. Additionally, it is important to identify if and how sites adapt standardized HIR implementation strategy by introducing their own, site-initiated implementation strategies and to assess the influence of these modifications on implementation outcomes [15]. To develop more generalizable recommendations for HIR implementation, it is important to examine HIR implementation across multiple sites due to the variation in facility-level implementation outcomes (e.g., heterogeneity in intervention fidelity and adoption rates) observed in multisite implementation efforts [16, 17].

Achieving rigorous, reproducible, and actionable examination often requires integrating multiple theories, models, taxonomies, or frameworks through a logic model [18–29]. The Implementation Research Logic Model (IRLM) supports this integration by explicitly mapping the relationships between key components: 1) determinant frameworks to identify contextual barriers and facilitators (e.g., Practical, Robust Implementation and Sustainability Model, Consolidated Framework for Implementation Research, Theoretical Domains Framework); 2) taxonomies to classify implementation strategies (e.g., Experts Recommendations for Implementation Change and Effective Practice and Organization of Care); 3) mechanisms of action explaining how strategies produce change; and 4) frameworks to evaluate implementation outcomes (Reach, Effectiveness, Adoption, Implementation, and Maintenance and Implementation Outcomes Framework). The IRLM illustrates how contextual determinants inform the selection of strategies, how those strategies operate through mechanisms, and how mechanisms contribute to specific implementation and effectiveness outcomes. This systematic mapping enables identification of provisional pathways, hypothesized sequences connecting strategies to outcomes via mechanisms, supporting better design, tailoring, replication, and scalability of implementation strategies [25, 30]. To guide these connections, tools such as Michie et al.’s behavior change technique-mechanism mapping provide structured approaches for linking strategies to their theoretic mechanisms of action and associated outcomes [31].

Accordingly, this study uses a convergent mixed-methods, multi-site case study design to develop an IRLM and examine HIR implementation by: 1) determining the extent of implementation per site, 2) systematically identifying contextual factors (barriers or facilitators) influencing HIR implementation, 3) systematically identifying site-initiated implementation strategies and their mechanism, and 4) linking factors, strategies, and mechanisms using the Implementation Research Logic Model to propose provisional pathways for HIR implementation in SNFs. Upon completion, the drafted IRLM will guide planning of additional HIR implementation efforts.

## Methods

This paper follows the reporting standards: Standards for Reporting Implementation Studies (StaRI) [32], Mixed Methods Reporting in Rehabilitation and Health Sciences (MMR-RHS) [33], Standards for Reporting Qualitative Research (SRQR) [34].

As part of a collection of efforts to enhance SNF rehabilitation through HIR implementation, this study employed a convergent mixed-methods multi-site case study design, following methods outlined by Yin [35] and Fetters [36]. This design enabled in-depth exploration of implementation across eight geographically diverse Veteran’s Health Administration (VHA) SNFs in the United States participating in a quality improvement initiative to implement HIR. It allowed us to capture variation in implementation outcomes and contextual factors. Convergent methods allowed integration and triangulation of quantitative and qualitative data to strengthen interpretation. A waiver of documentation of informed consent was obtained from both the VA Research Administration Office and the University of Colorado Anschutz Medical Center Institutional Review Board (IRB number 21–4637).

Sites were recruited through VHA email lists targeting medical and rehabilitation directors. Sites were included once they confirmed consistent admission of short-term stay patients and committed to facilitating participation of full-time and part-time rehabilitation clinicians (i.e., physical therapists, physical therapy assistants, occupational therapists, occupational therapy assistants) in an 18-week HIR implementation program. This program included a standardized, research-led multicomponent implementation strategy, participation in focus groups, and questionnaire completion. Site demographics are presented in Table 1.

**Table 1:** Site demographics

	Site A	Site B	Site C	Site D	Site E	Site F	Site G	Site H
**Rehabilitation Staff Size**								
	5	4	2	4	8	12	4	6
**Discipline**								
PT	2 (40%)	2 (50%)	1 (50%)	1 (25%)	2 (25%)	3 (25%)	1 (25%)	2 (33%)
PTA	1 (20%)	1 (25%)	0	1 (25%)	1 (13%)	1 (8%)	2 (50%)	1 (17%)
OT	2 (40%)	1 (25%)	1 (50%)	1 (25%)	3 (38%)	3 (25%)	1 (25%)	2 (33%)
OTA	0	0	0	1(25%)	1 (13%)	1 (8%)	0	1 (17%)
KT	0	0	0	0	0	4 (33%)	0	0
Other	0	0	0	0	1 (13%)	0	0	0
**Specialty Certification Indicated**								
	0 (0%)	2 (50%)	1 (50%)	0 (0%)	2 (25%)	7 (58.33%)	1 (25%)	0 (0%)
**Years in practice**								
Mean (SD) Range	15.60 (12.19) 5–36	22.50 (7.32) 15–31	31.50 (3.54) 29–34	11.00 (11.63) 1–27	17.25 (11.13) 5–30	15.58 (9.14) 3–30	22.50 (9.57) 10–30	12.42 (7.05) 5–24
**Years in current role**								
Mean (SD) Range	7.50 (5.57) 2–15	4.50 (2.65) 1–7	18.50 (9.19) 12–25	9.50 (9.75) 1–23	7.75 (9.31) 1–29	9.29 (4.75) 3–18	17.5 (10.34) 6–30	5.88 (9.03) 0.25–24
**Gender**								
Male	1 (20%)	1 (25%)	0	2 (50%)	2 (25%)	3 (25%)	1 (25%)	1 (17%)
Female	4 (80%)	3 (75%)	2 (100%)	2 (50%)	6 (75%)	9 (75%)	3 (75%)	5 (83%)
**Race**								
Am Ind	0	0	0	0	0	0	0	0
Asian	1 (20%)	1 (25%)	0	0	0	0	0	5 (83%)
Black	0	0	1 (50%)	0	0	2 (17%)	0	0
White	4 (80%)	2 (50%)	1 (50%)	4 (100%)	8 (100%)	10 (83%)	3 (75%)	0
Multiracial	0	0	0	0	0	0	0	1 (17%)
Other or unknown	0	1 (25%)	0	0	0	0	1 (25%)	0
**Ethnicity**								
Hispanic	0	3 (75%)	0	2	1 (13%)	1 (8%)	0	0
Non-Hispanic	5 (100%)	1 (25%)	2 (100%)	2 (100%)	7 (87%)	10 (83%)	3 (75%)	6 (100%)
Unknown	0	0	0	0	0	1 (8%)	1 (25%)	0

All sites are part of the Veterans Health Administration

*PT* Physical therapist, *PTA* Physical therapy assistant, *OT* Occupational therapist, *OTA* Occupational therapy assistant, *KT* Kinesiotherapist, *Am Ind* American Indian

### High-intensity resistance rehabilitation

HIR, detailed elsewhere [8, 37], enhances patient function by applying high-intensity dosing principles, such as an 8-repetition maximum or 80% task completion, across therapeutic interventions like exercise; activity of daily living simulation; and gait, balance, and transfer training (Additional file 1). Clinicians in rural VA skilled nursing facilities were trained to tailor HIR principles to patient goals, progressively increasing intensity each session by adjusting external load, prolonging the eccentric phase of movements, or incorporating environmental barriers and cognitive demands.

### Research-led multicomponent implementation strategy

To support scalability and consistency, the research team prospectively developed and delivered a standardized implementation strategy. To ensure this strategy was empirically sound, partner-informed, and contextually appropriate, we followed the steps of Implementation Mapping, a systematic approach for selecting strategies to address contextual factors [27]. Briefly, a panel of clinicians and clinician-researchers with experience implementing HIR into SNF triangulated contextual factors from their own experience and a literature review, then used theory and evidence to select or develop strategies targeting those factors. This process served as an initial needs assessment in lieu of a site-level contextual analysis. Full details of this process are reported elsewhere [38].

**Fig. 1:**
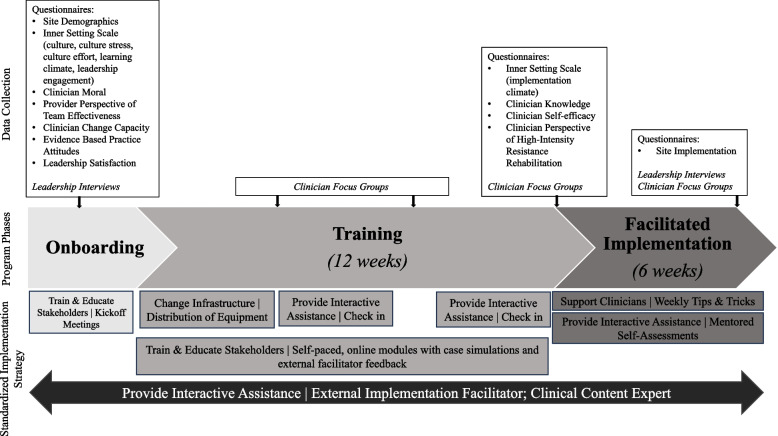
Program Timeline Including Data Collection and Standardized Implementation Strategy Components. Training involves clinicians completing online High-Intensity Resistance Rehabilitation modules. Facilitated Implementation included clinician mentorship and reminders. Details on implementation strategy components per phase provided in Additional file 2

The standardized, research-led multicomponent implementation strategy received by all sites included: 1) education and training of clinicians and leadership, 2) infrastructure change through the distribution of equipment, 3) interactive assistance through access to an external implementation facilitator and clinical content expert, and 4) clinician support through regular emailed tips and tricks (Fig. 1). Notably, the external implementation facilitator was available to address any site-specific needs. Additional file 2 further details the standardized implementation strategy following established reporting procedures. Though engagement with the standardized strategy was encouraged as part of program participation, we observed that sites adapted strategy elements and enacted their own strategies in response to local needs, prompting our retrospective assessment. While formal, prospective evaluation of this standardized strategy is reported elsewhere (currently under review), this paper focuses on describing and understanding site-initiated strategies and adaptations. This approach enables a more comprehensive understanding of how standardized and emergent strategies function in real-world implementation and informs the development of practical recommendations for future HIR implementation.

### Implementation research logic model

**Fig. 2:**
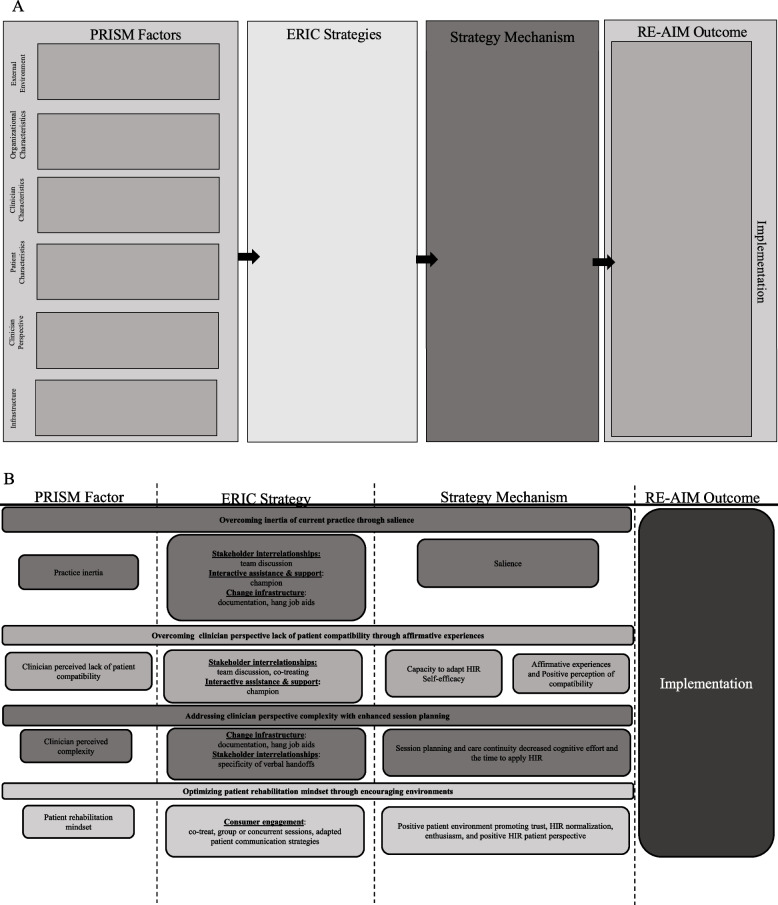
Implementation Research Logic Model. **A** High-Intensity Resistance Rehabilitation Implementation Research Logic Model (IRLM) template linking Practical Robust Implementation and Sustainability Model (PRISM), Expert Recommendations for Implementing Change (ERIC), strategy mechanisms, and Reach, Effectiveness, Adoption, Implementation, Maintenance (RE-AIM) to guide data analysis. **B** Composite IRLM Identifying Provisional Pathways for High-Intensity Rehabilitation Implementation (HIR). Four provisional pathways for high-intensity rehabilitation implementation identified via cross-site analysis linking elements of the Implementation Research Logic Model including shared contextual factors, strategies, mechanisms, and relevant impact on high-intensity resistance rehabilitation implementation

Our Implementation Research Logic Model (IRLM) included the Practical Robust Implementation and Sustainability Model (PRISM), its Reach, Effectiveness, Adoption, Implementation, and Maintenance (RE-AIM) outcomes framework, and the Expert Recommendation for Implementing Change (ERIC) (Fig. 2a). PRISM offers a comprehensive model of contextual factors hypothesized to influence implementation outcomes, considering characteristics and perspectives of organizations, staff, and beneficiaries of evidence-based interventions. PRISM guided the identification of contextual factors influencing HIR implementation [24, 39–42] (Table 2). ERIC helped organize and describe site-initiated implementation strategies employed in addition to the standard research-led strategy [21, 43, 44] (Table 2).

**Table 2:** Study frameworks, constructs, and associated data sources

**Framework**	**Use in study**	**Construct or Domain**	**Quantitative Questionnaires** *Psychometric Properties*	**Questionnaire Scoring**
PRISM	PRISM guided identification and description of factors influencing site implementation Focus group and interview guides and a deductive coding structure were developed according to PRISM constructs Quantitative questionnaires were selected to represent PRISM constructs	Organizational Characteristics		
		Culture	Inner Setting Scale [39] (culture, culture stress, culture effort, learning climate) *Acceptable Internal Consistency (Cronbach’s* α > *0.70)* *Good Construct Validity (CFI* > *0.90, RMSEA* < *0.08)*	1–5^a^
		Leadership	Inner Setting Scale [39] (leadership engagement) *Good Internal Consistency (Cronbach’s* α = 0.87) *Good Construct Validity (CFI* = *0.95, RMSEA* = *0.06)* 1-item study-derived questionnaire assessing satisfaction with leadership	1–5^a^ 1–3^a^
		Morale	Self-Defined Burnout Measure [40] *High Specificity (94.7%)* *Low Sensitivity (50.4%)* *Good Concurrent Validity (qualitatively supported)* Utrecht Engagement Scale [42] *High Reliability (rhoMS* = *0.85*) *Satisfactory Scalability [Construct Validity (Mokken H* > *0.50)]* 4-item study-derived work satisfaction questionnaire	1–5^b^ 0–6^a^ 1–5^a^
		Team Effectiveness & Communication	Provider Perspective of Team Effectiveness Questionnaire [44] *Good Internal Consistency (Cronbach’s α* = *0.91)* *Good Responsiveness (differences between high low functioning teams p* < *0.001)* *Good Face Validity (established through expert feedback)* *Good Content Validity (confirmed by expert opinion)* *Good Construct Validity (Known-group technique with significant differences by length of time in team p* = *0.025)* 3-item study-derived questionnaire assessing communication satisfaction	1–6^a^ 1–3^a^
		Clinician Characteristics		
		Knowledge	Post-training assessment of didactic knowledge	0–100^a^
		Self-Efficacy	Post-training assessment of confidence in ability to integrate high-intensity resistance rehabilitation into clinical care	1–5^a^
		Evidence Based Practice Attitude	Evidence Based Practice Attitudes Scale (EBPAS) [45] *Acceptable Internal Consistency (Cronbach’s α* = *0.79)* *Acceptable Construct Validity (CFI 0.93; RMSEA 0.067)*	0–4^a^
		Implementation and Sustainability Infrastructure		
		Change Capacity	5-item study-derived questionnaire to identify capacity specific to implementing high-intensity resistance rehabilitation including resources, time, leadership support, communication channels, perceived need	1–5^a^
		Implementation Climate	Implementation Climate (Inner Setting Scale) [39] *Acceptable Internal Consistency (Cronbach’s α* = *0.84)* *Acceptable Construct Validity (RMSEA* = *0.07)*	1–5^a^
		Clinician Perspectives of High-Intensity Resistance Rehabilitation		
		Perspective of Intervention	Perceived Characteristics of Intervention Scale (PCIS) [46] *Good Internal Consistency (Omega h* = *0.91)* *Good Construct Validity (CFI* = *0.957, RMSEA* = *0.040)* • Positive Sub Score • Risk Sub score	1–5^a^ 1–5^b^
RE-AIM	RE-AIM represents salient implementation outcomes. For this study, implementation was evaluated through a quantitative measure triangulated with qualitative data	Implementation	Provider Report of Sustainment Scale (PRESS) *Good Internal Consistency (Cronbach’s α* = *0.95)* *Acceptable Face and Construct Validity (established by expert opinion and correlations with related variables: Sustainment Climate Scale, Sustainment Leadership Scale, Evidence-Based Practice Attitude Scale)*	0–4^a^
ERIC	ERIC assisted in description of a priori implementation strategies along with identification and description of any actions taken by sites to encourage implementation. Focus group and interview guides and a deductive coding structure were developed according to ERIC			NA

*PRISM* Practical Robust Implementation and Sustainability Model, *ERIC* Expert Recommendations for Implementing Change, *RE-AIM* Reach Effectiveness Adoption Implementation Maintenance, *CFI* Comparative Fit Index, *RMSEA* Root Mean Square Error of Approximation

^a^indicates higher scores as more favorable

^b^indicates higher scores as least favorable

### Data collection

Following convergent mixed-methods procedures, qualitative and quantitative data collection assessing elements of the IRLM included questionnaires, semi-structured leadership interviews, and clinician focus groups [45] occurred simultaneously (Fig. 1). We used Research Electronic Data Capture (REDCap) [46, 47], hosted at University of Colorado, to collect and manage questionnaire data. A case study database developed in Microsoft Excel managed the multiple data sources [35, 45, 48].

#### Quantitative questionnaires

At the time of this work, no established PRISM-informed questionnaires existed. Therefore, we conducted a literature search to identify validated questionnaires aligned with PRISM constructs. Validated tools were used when available and relevant, with some items adapted to fit our context. Where gaps in existing tools remained, we developed study-specific items to ensure key constructs were assessed (Table 2). Questionnaires are grouped by those that evaluate implementation and those that assess PRISM constructs. Individual questionnaires are attached in additional file 3.

1. *Questionnaires of site implementation *
  Due to COVID-related restrictions and limited access to electronic medical records, remote measurement of *site implementation* was conducted through a clinician questionnaire- the Provider Report of Sustainment Scale (PRESS), which consists of three questions on a 0 to 4 scale with higher scores indicating greater extent of implementation [49]. The PRESS was selected as a practical measure aligned with RE-AIMs definition of implementation, focusing on the consistency of intervention delivery [50]. While the scale’s title mentions “sustainment”, its questions assess the extent to which staff integrates an evidence-based practice into routine practice, reflecting implementation consistency [49].
2. *Questionnaires of PRISM contextual factors*
  *Organizational Characteristics*, including *culture, leadership, morale, and team effectiveness*, were assessed. The Inner Setting Scale [51], a valid and reliable measure of organizational culture, climate, and leadership [51], was employed to gauge organizational culture, climate, and leadership. Leadership communication satisfaction was evaluated through a single item, Likert question. Morale was measured via three surveys: the Self-Defined Burnout Measure [52, 53] for clinician burnout, the Utrecht Engagement Scale (UWES-3) [54] for work engagement, and a study-specific 4-item questionnaire for overall work satisfaction. Given that SNF rehabilitation relies on interdisciplinary team efforts and cohesiveness [55], team effectiveness was assessed using adapted items from the Provider Perspective of Team Effectiveness Questionnaire [56] (Provider-PTE), focusing on coordination, cohesion, problem-solving, and focus along with queries regarding communication patterns and satisfaction.
  *Clinician Characteristics* of interest included *knowledge* regarding HIR, *self-efficacy* in implementing HIR, and general *evidence-based practice attitudes*. Didactic knowledge and self-efficacy were measured using study-derived tools. The Evidence Based Practice Attitude Scale [57] measured clinician attitudes towards general evidence-based practice.
  *Implementation and Sustainability Infrastructure* was assessed using the *implementation climate* subscale of the Inner Setting Scale [51], and a 5-item questionnaire developed to comprehensively measure *change capacity* including existing resources, leadership support, and communication channels required for HIR implementation.
  *Clinician Perspective* of HIR was measured using the Perceived Characteristics of Intervention Scale [58]. This scale is largely based on Rogers Theory of Innovation with constructs added by Greenhalgh et al. [59], and results can be presented across two scores: positive perspectives and perspective of risk [60].

#### Qualitative data collection

A post-positivist theoretical framework [61], combined with the goal of integrating quantitative and qualitative data for actionable findings, guided a deductive emphasis within our hybrid inductive-deductive approach [62–64]. Rigor was ensured using methods by Morse, et al. including responsiveness, verification, methodological coherence, theoretical and adequate sampling, and an active analytic stance [65]. Semi-structured interview and focus group guides were developed based on study frameworks (i.e., PRISM, ERIC, RE-AIM) and current study aims, focusing on exploring implementation examples, exploring the presence and influence of contextual factors, identifying site-led strategies, and exploring strategy influence on implementation. Guides were iteratively refined after team piloting. Sample questions included: *Please describe your experience implementing high-intensity rehabilitation* and *Tell me about how your team functioned during implementation* (exploring implementation extent); *Of the challenges you described, which stand out to you as being the most influential?* and *Of the factors you described as supporting or facilitating the program, which stand out to you as being most influential?* (exploring factors influencing implementation), and *As you think about your workflows and processes, have they changed as a result of implementing the program? How?, What processes, if any, did you or your team adopt to assure ongoing implementation or to overcome some of the challenges?, What are your general impressions of the support provided during this implementation and how were those supports helpful or not helpful?,* and *What support, if any, was provided by leadership during implementation and how did that influence your ability to implement* (exploring site-led strategies and strategy mechanisms). Following purposive sampling, all participants [leaders (i.e., Medical Directors, Directors of Rehabilitation) and clinicians] were recruited for interviews and focus groups as part of their agreement to participate in the quality improvement initiative. Two leadership interviews and four clinician focus groups occurred per site at various stages of the initiative (Fig. 1). Interviews and focus groups lasting 30–60 min were conducted on Microsoft Teams during clinic hours by two research personnel also serving as external implementation facilitators. Post-data collection, a debrief form was completed by the research personnel.

### Data analysis

To determine implementation extent and describe potential influencing factors, descriptive statistics [mean (SD), median (IQR), or frequency (percentage) where appropriate] of PRESS and PRISM constructs per site were calculated using SAS OnDemand for Academics (SAS Institute Inc. 2023). Also to determine implementation extent and identify PRISM constructs along with site-initiated strategies and strategy mechanisms (how strategies influenced implementation), interviews and focus groups were recorded, transcribed verbatim, checked for accuracy, uploaded to Atlas.ti Windows (Version 23.2.2) [66] qualitative management software, and analyzed using a hybrid deductive and inductive coding, thematic matrix analysis, and narrative case summaries [67, 68]. A team of four researchers, two of which facilitated data collection, doubled coded all transcripts. Consensus meetings were held with the study team for discussing the valence (positive/facilitator; negative/barrier) and salience (strength) of identified contextual factors and within- and across-case analysis including narrative case summaries and provisional pathways [35]. Necessary steps were taken to minimize bias and subjectivity within the analysis team including triangulation across four researchers and reflexivity.

#### Mixed method analysis

Data mixing followed the framework of mixed-methods case study [36] and occurred across multiple steps using joint displays, which enabled both within- and cross-site analysis. First, to determine extent of HIR implementation, quantitative (PRESS) and qualitative data were merged via joint display and evaluated for congruence and expansion [36, 69, 70]. Sites were classified into quartiles based on PRESS scores: the upper quartile indicated high extent of implementation, the middle quartile denoted moderate extent, and the lower quartile suggested comparatively lower extent of implementation [71].

Second, to identify trends in contextual factors and site-initiated strategies, we generated three matrix-based heat maps, an approach known to be effective in small sample studies [72]. These included a: 1) quantitative heat map, 2) qualitative heat map, and 3) site-initiated strategy heat map. For the quantitative matrix, we applied an exploratory method due to the absence of established cut-off course for our selected questionnaires. Data were organized into a matrix with sites as rows and PRISM constructs as columns. Questionnaire scores were divided into quintiles, and a 5-color gradient was applied using Microsoft Excel programing to visually reflect the range of scores. Rows were ordered from highest to lowest implementation (based on PRESS scores), allowing visual identification of patterns in barriers and facilitators by site performance.

The qualitative matrix mirrored this structure. Sites (rows) were again ordered by implementation extent, and columns represented PRISM constructs. A 5-color gradient was applied based on the presence or absence, valence, and salience of qualitative themes, representing a range of significant facilitators to significant barriers. This enabled visual comparison of qualitative patterns by site performance.

For the site-initiated strategy matrix, rows represented sites and columns depicted site-initiated implementation strategies. A 2-color scale was applied indicating presence or absence of the strategy.

Third, all heat maps were laid side-by-side to explore convergence across data types and identify cross-site patterns of contextual factors and site-driven strategies that may explain variation in implementation success.

**Fig. 3:**
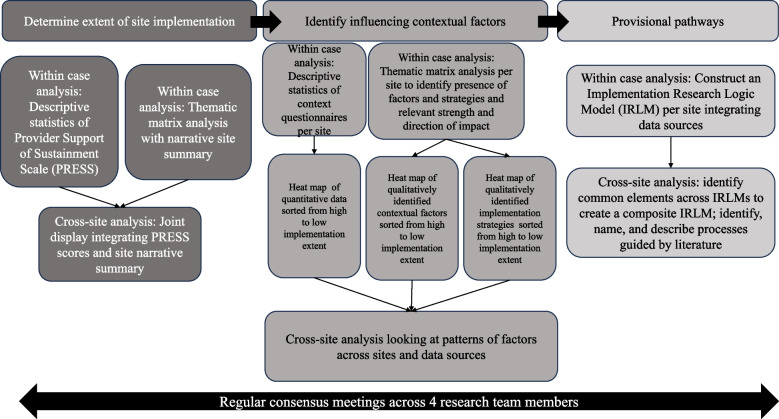
Data Analysis Steps

Proposal of provisional implementation pathways occurred in three steps. First, an IRLM was constructed for each site, incorporating site-specific data, including participant-reported contextual factors, implementation strategies, and mechanisms described by the participants. Second, shared elements across site-specific IRLMs were synthesized into a composite IRLM for HIR implementation in SNFs. Third, we used this composite IRLM to identify, name, and describe provisional pathways. These pathways were grounded in participant descriptions and further refined using Michie et al.’s approach to linking behavior change techniques and theoretical mechanisms of action [31] (Fig. 3).

## Results

### Site-level implementation

Concordance was observed in merging data strands describing implementation (Table 3). PRESS scores ranged from 3.75 (0.17) to 2.33 (0.67) on a 4-point scale, with all sites implementing HIR to at least a “moderate extent” [49].

**Table 3:** Mixed methods findings representing site-level implementation

	**Site D**	**Site C**	**Site E**	**Site F**	**Site G**	**Site B**	**Site H**	**Site A**
PRESS Score^a^	3.75 (0.17)	3.33 (0.47)	2.88 (0.17)	2.63 (0.90)	2.58 (0.50)	2.58 (0.96)	2.44 (0.50)	2.33 (0.67)
Quartile	Upper		Middle				Lower	
Qualitative	Thick, rich examples provided across clinical roles, rehabilitation interventions, and patient populations	Thick, rich examples provided across clinical roles, rehabilitation interventions, and patient populations	Thick, rich examples provided by PT/OT; moderate volume and specificity of examples provided by PTA/OTA	Limited volume and specificity of examples across team	Limited examples provided by assistant team (PTA, OTA)	Limited examples provided by OT team	Minimal examples provided across team	Minimal examples provided across team
Meta-inferences	Concordance identified across data strands for implementation. Higher-implementing sites demonstrated collective team implementation while lower-implementing sites lacked collective team implementation. Sites in the middle quartile appeared to have a clinician group (either occupational therapy or assistants) that implemented to a limited extent							

^a^Mean (SD). Sites presented in order of highest to lowest score on a 4-point scale

Qualitative data revealed implementation examples from all sites; however, variations were observed in both the nuance and volume of these examples. Variations included how HIR was adapted across different rehabilitation interventions to accommodate diverse patient populations; the depth, specificity, and variety of examples provided; and differences in the representation of team members contributing examples. Higher-implementing sites (upper quartile PRESS score) provided a greater number of nuanced implementation examples, with input from the entire team, indicating more comprehensive implementation. Conversely, lower-implementing sites (lower quartile PRESS score) provided fewer implementation examples, which were less detailed, specific, and varied, and often lacked contributions from the full team. For instance, one lower-implementing site provided specific implementation examples from supervising therapists but fewer from therapy assistants, while another site had occupational therapy team members who did not contribute examples.

### Contextual factors

**Fig. 4:**
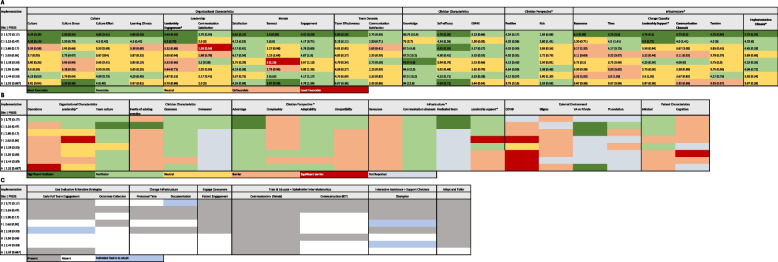
Heat Maps. **A** heat map of quantitatively sourced contextual factors. **B** heat map of qualitatively identified contextual factors. **C** heat map of site-initiated implementation strategies

*Clinician perspectives, site infrastructure,* and *general satisfaction with leadership engagement* were identified as key contextual factors shared across data types delineating the contrast between higher-implementing and lower-implementing sites (Fig. 4a, b). Quantitatively measured contextual differences between higher and lower implementing sites can mostly be observed in the organizational characteristics (leadership engagement), infrastructure (resources, time, leadership support, necessary communication channels, tension for change, implementation climate), and clinician perspective domains. A consistent pattern was observed, where higher-performing sites scored in the upper quintiles, while lower performing sites scored in the lower quintiles for tools representing these constructs (Fig. 4a).

Qualitative data provided deeper insights into the nuances of clinician perspectives highlighting perspectives of *HIR complexity and patient compatibility* as delineating constructs (Fig. 4b). These data also expanded our understanding of delineating factors that were not measured quantitatively, specifically shedding light on the impact of *patient characteristics* (rehabilitation mindset). Across most sites, strong facilitators of HIR implementation included: 1) the environment of the Veterans Health Administration encompassing clinician autonomy, clinician access to medical records, models of care, and Veterans’ experience with physical exertion; 2) a collaborative and patient-centered interdisciplinary team culture; 3) clinician openness to change; 4) supportive infrastructure for sharing best practices and critical patient details; and 5) clinician recognition of HIR adaptability and value through its ability to improve functional outcomes more quickly than prior rehabilitation approaches. Shared barriers comprised of external factors including COVID-related challenges and SNF stigma, inertia of existing practice, patients with low cognitive status, and resource constraints (e.g., limited resistance equipment and space) (Fig. 4b and additional file 4).

### Site-initiated implementation strategies and their mechanism

All sites attempted to overcome barriers by initiating their own implementation strategies alongside the standard research-led strategy (Fig. 4c and additional file 5). Site-initiated strategies were inductively identified through participants interviews and focus groups, then mapped to ERIC thematic clusters. These identified: 1) evaluative and iterative strategies, 2) infrastructure change, 3) patient engagement, 4) stakeholder interrelationships, 5) interactive assistance for clinicians, and 6) adaptation and tailoring. Among these, stakeholder interrelationships, notably shifting rehabilitation team communication to include HIR discussions, emerged as the most utilized strategy. This was followed by adapting HIR to fit patient contexts and engaging patients. Clinicians and leaders reported these strategies influenced implementation by: 1) enhancing clinician positive perspective, 2) creating a positive patient environment, 3) providing early affirmative experiences, 4) decreasing cognitive effort through session planning and execution, 5) enhancing adaptation capacity and confidence, 6) optimizing care continuity, 7) and optimizing HIR salience (clinician awareness and perception of importance). Higher implementing sites reported higher use of site-initiated strategies, in addition to the research-led strategies. Strategies that differentiated higher from lower implementing sites included patient engagement, adaptation, presence of an informal champion, and early team engagement.

### Provisional implementation pathways

Using qualitative findings of strategy mechanisms or “how” the research-led and site-led implementation strategies influenced shared contextual factors and implementation, we constructed a single IRLM representing implementation across all sites that linked barriers to ERIC strategies which allowed development of provisional pathways contributing to implementation: 1) overcoming inertia of current practice through HIR salience, 2) overcoming clinician concerns of patient compatibility through affirmative experience, 3) addressing clinicians perspective of complexity with session planning, and 4) optimizing patient rehabilitation mindset through encouraging environments (Fig. 2b).

1. *Overcoming inertia of current practice through HIR salience*
  When clinicians experienced the barrier of inertia of current practice, sites initiated strategies that improved implementation by making HIR more salient. This included frequent team discussion of HIR, hanging of job aids (a resource offered by the research team as a quick reference guide to patient screening and dosing principles), changes in documentation, and/or emergence of an informal champion.
  *The job aids…where to position them in the gym or giving them out individually to each of us so that we always got a visual prompt for, okay, are you checking this? Are you trying these things? Are you progressing through this? Not only are we accountable to each other but throughout the day we’ll visually have a reminder of continuing to incorporate that and push our patients as well*. (PT 7) *…even small things like making it part of our consult template. “Is this patient potentially appropriate for high intensity rehab, methodology” to just continue to keep that in their mindset that, “Hey, I’ve got to keep remembering that this is how we do things.” after a period of time, I would hope that this is what comes first when you think about taking care of a patient*. (Leader 6)
2. *Overcoming clinician perceived lack of patient compatibility through affirmative experiences*
  Clinicians and leaders described how team discussion, co-treating, and the emergence informal champions may have influenced a shift in their perspectives, leading to greater perceived compatibility between HIR and patients. These actions improved implementation through support and mentorship, boosting clinicians’ confidence and ability to adapt HIR to diverse patient contexts. Experiencing this support, along with observing their enhanced HIR delivery skills and positive patient responses, created affirmative experiences for clinicians.
  *I almost saw a little pullback from her when she seemed to think this is mostly a PT thing. I talked to her, and was like this is universally applicable. You could break it down to even hand squeezes. The level of anything that needs strengthening, we just need to do on different parameters to really get the optimal strength gain. I think she was seeing it more and more as it was going along and I see them utilizing it more now.* (Leader 4)
3. *Addressing clinician perspective of complexity with session planning*
  The barrier of perceived HIR complexity was addressed by visually displaying job aids and refining specificity of documentation, verbal patient handoffs, and team discussion. These strategies decreased the cognitive effort required to implement HIR through more efficient care continuity, session planning, and identification of intervention ideas.
  *Here we have more discussion as a team…we have a great team as far as the creative really goes, we can come up with some crazy ideas. *(PT 1)
4. *Optimizing patient rehabilitation mindset through encouraging environments*
  Clinicians targeted patient rehabilitation mindset and engagement with HIR by using strategies that created an environment of trust, peer normalization, and enthusiasm. These strategies included co-treating, group or concurrent sessions, and adapted patient communication.
  *I was explaining it to them as we went. “I really want to work with you at a higher challenging level so that we can see results a little bit quicker. If I just keep having you push through, doing three sets of 10 and you're not seeing any benefit in it, then, it's not benefiting you."They really did seem to buy in. It seemed like there was a little spark that came to life in them as they were finding those levels of challenge. So I think they really did enjoy it at the end*. (PTA 2)
  *…that’s very helpful to provide that education…. to remember to instead of just you monitoring the vitals to bring them [patients] into it. *(OT 5)

## Discussion

This study sought to enhance understanding of HIR implementation in SNFs through a convergent mixed-methods, multi-site case study approach as part of a quality improvement initiative using a standardized, research-led implementation strategy. By retrospectively evaluating implementation using an IRLM, we systematically identified influential contextual factors, site-initiated implementation strategies, and proposed four provisional pathways of HIR implementation in SNFs to enhance the current implementation strategy and inform future implementation efforts.

Though we used a well-established approach of implementation strategy development, [27] this study identified several opportunities to enhance HIR implementation efforts. Previous research underscores the significance of organizational systems, team dynamics, patient and therapist self-efficacy, and perspectives of intervention effectiveness in driving rehabilitation practice change in SNFs [9]. This study adds specificity to these influencing factors including perspectives of HIR complexity and patient compatibility, patient factors including clinician-reported patient agreement, inertia of existing practice, and infrastructure. Additionally, we offer strategies to better target these factors.

Many clinical teams were hesitant to use HIR because they perceived it to have low compatibility with patient needs and abilities and believed that its high delivery complexity would require more time for implementation. Based on the research team’s prior HIR implementation experience, these perspectives were anticipated. Efforts to address this included training with worked examples, efficiency steps, and patient testimonials, but these measures were insufficient to overcome this barrier. Findings suggest clinicians may benefit from direct mentoring tailored to their unique patients to build confidence in efficiently adapting HIR. They also require early implementation support through observational learning, social models, and direct experience of successful outcomes for themselves and their patients. This will enhance positive outcome expectations, which is important for behavior change [73–75]. This can be facilitated by a Champion, co-treating, team idea sharing, or setting both short- and long-term realistic implementation goals [76]. Also to address complexity and perceived increased time requirement for implementation, sites enhanced their specificity of documentation and patient handoffs. Future HIR implementation efforts should provide better support for adapting HIR to diverse patient presentations through worked examples, offer more mentored adaptation practice, empower Champions with mentoring skills, and improve patient handoff processes.

Implementation models emphasize patient characteristics, with nearly half of rehabilitation implementation studies citing patient needs as influential [77]. Clinicians identified patient attributes, such as cognition, willingness to engage in rehabilitation, mindset, affect, and self-efficacy as notable barriers to HIR implementation. Therapists indicated that patient’s previous rehabilitation experience and anticipated discharge destination influenced patient mindset, affect, and willingness to engage. Interestingly, clinicians from higher-implementing sites viewed patient characteristics as less of a barrier, raising questions about differences in patient case mixes or their ability to overcome patient-related challenges. Some sites worked to overcome this patient-related challenge by adapting communication and shifting their care model to include more co-treats (more than one clinician simultaneously providing rehabilitation to a patient) and concurrent or group therapies (one clinician providing rehabilitation to more than one patient simultaneously), aiming to foster patient trust, peer encouragement and enthusiasm, and establish HIR as a social norm. Although these approaches may not be feasible everywhere, identifying their impact helps guide the selection of other strategies to enhance patient engagement by building trust and establishing social norms. To better understand patient characteristics as an influencing factor and determine necessary implementation strategies, patient perspectives on HIR are needed.

The inertia of existing practices significantly influences implementation of other practices. Our results indicate that multiple approaches can be employed to enhance HIR salience to overcome this momentum and thus improve implementation of HIR. These approaches encompass engaging in regular team discussion or “huddles”, the presence of a Champion, or changing site infrastructure including documentation prompts or the visual display of job aids. Another consideration may include incentives and recognition including the integration of HIR applications into annual performance reviews [78, 79]. Furthermore, to bolster salience and implementation of HIR, a behavioral economics perspective may prove beneficial. This involves a deeper understanding of clinician decision-making processes and heuristics, along with using strategies like nudging or adjusting choice architecture for clinicians. For instance, structuring the physical environment to ensure that necessary equipment is prominently displayed and easily accessible can facilitate HIR implementation [80].

Infrastructure plays an important role in implementation efforts [41]. Key subconstructs for HIR implementation included: 1) resources (physical and time), 2) leadership support and acknowledgement, 3) channels to share best practice and critical patient details, and 4) shared expectation, prioritization, and tension for change—defined as the perceived urgency to adopt a new practice due to recognition of a care gap or dissatisfaction with status quo [59]. Higher-implementing sites employed more site-initiated implementation strategies, suggesting their infrastructure may be better suited for strategy deployment. To implement HIR to a high extent, efforts should focus on fostering this constructive infrastructure. Specifically, higher-implementing sites had leaders who balanced oversight and autonomy, remained aware of program progress, provided individualized acknowledgment and mentorship, and were sensitive to clinician needs and preferences. Sites lacking such leadership indicated its need, while leaders requested guidance on how to support their clinical teams. These findings are supported empirically [81–84] and by the many implementation frameworks positing that leadership serves a critical role in implementation across diverse fields [18, 39, 85]. Guided by our findings and those from a recent review on leadership in implementation [84], future efforts will develop and pilot strategies targeting leadership to optimize implementation.

To further enhance implementation strategy design and impact, it is important to understand how strategies function through specific mechanisms. We conceptually linked contextual barriers and facilitators to strategy functions and their mechanisms of action. These mechanisms, including salience, confidence, and skill development, were informed by participant experience and prior literature [31]. While we did not apply a single unifying theory, this approach integrates theory, evidence, and clinical experience. Future work may benefit from prospectively applying mechanism mapping approaches to strengthen theoretical clarity and transferability.

The interconnectedness of PRISM constructs adds complexity to interpretation of our findings, but it also underscores the need for critical next steps. This includes investigating key factors to determine if they are influenced by deeper, underlying issues. If so, these issues may need to be addressed due to their widespread impact. For example, initial hesitancy from all clinicians was fueled by low perspectives of HIR compatibility with typical SNF patient populations. This perspective may be fueled by an underlying level of stigma including ageism. Though no empirical work regarding stigma in SNF could be found, literature demonstrates how ageism can negatively impact quality of care provided [86] and is harmful to older adult health and well-being [87]. Specifically in rehabilitation, ageism leads to under-dosing of exercise interventions, reduced attention to patients'concerns, a lack of empathy, and a decreased likelihood of promoting physical activity [88–90]. This contrasts with the American Physical Therapy Association’s guidelines for the Choosing Wisely campaign, which include the recommendation,"Do not prescribe underdosed strength training programs for older adults” [91].

### Limitations

This work used a novel convergent mixed-methods approach but has limitations. First, team implementation was measured via self-report survey and not actual fidelity, a core element of implementation. Though the survey was validated, observation and chart-audit likely more accurately represent implementation. Observation was not feasible during the study period due to the COVID-19 health crisis. Chart-audit does not accurately capture implementation fidelity (e.g., target HIR dosing delivered) given documentation insufficiency. Future work will include both custom EMR fields with chart audits and observation to measure adoption and implementation. Second, due to difficulty scheduling, a full clinical team was not present at all focus groups; thus, findings may not represent the experience and perspectives of the entire clinical team. Third, there are no validated surveys to assess PRISM constructs. We used PRISM to select validated survey tools developed from other theories and frameworks, and to guide development of custom surveys. This may result in slight misrepresentation of PRISM constructs. This is, however, a known limitation in implementation research across many determinant frameworks, and we addressed this issue by using PRISM literature to guide qualitative data collection and a hybrid inductive/deductive analytical approach to allow for breadth of contextual factor identification. Finally, the generalizability of our findings beyond the VHA is somewhat limited. Given the sites’ perceived contextual differences between the VHA and the private sector influence implementation, one must take caution when applying these findings to sites external to the VHA.

Despite limitations, to our knowledge this study is the first in SNF implementation to rigorously report strategies and their connection to contextual factors and implementation outcomes, identifying provisional pathways for effective implementation. These results help understand not only what strategies are needed for HIR implementation in SNFs, but why they are necessary, what they target, and how they influence implementation extent. Collectively, these findings paired with tools published since this study’s execution including iPRISM [92] will aid future strategic decision-making and resource allocation.

## Conclusion

In conclusion, this multi-site case study identified contextual factors and site-initiated implementation strategies across eight sites that implemented HIR. This facilitated the creation of provisional pathways to consider when implementing HIR in other contexts and highlights shortcomings of our current strategy. Results guide refinement of a current HIR implementation strategy to better target salient influential factors and implementation pathways. To improve HIR implementation, future work must: 1) target infrastructure, including leadership support and communication channels, inertia of current practice, and clinician experience of patient agreeability to HIR as well as perspectives of HIR complexity and patient compatibility, and 2) seek to understand patient perspectives and experiences. Implementation strategies to consider include revision of current clinician education to better address clinician perspectives, formalization of team discussion and care continuity, patient engagement, a leadership toolkit, and identification and training of champions. The intent is for these strategies to promote HIR salience, clinician adaptation capacity, affirmative experiences and positive outcome expectation, efficiency with session planning, and a positive patient environment of trust and social normalization of HIR.

## Supplementary Information

Additional file 1. Description of High-Intensity Resistance Rehabilitation.

Additional file 2. Description of the Standardized Multicomponent High-Intensity Resistance Rehabilitation Implementation Strategy.

Additional file 3. Questionnaires.

Additional file 4. Qualitatively Identified PRISM Constructs and Factors by Site Implementation Extent.

Additional file 5. Site-Initiated Implementation Strategy Description and Mechanisms.

Additional file 6. StaRI Checklist.

## Data Availability

The datasets used and/or analyzed during the current study are available from the corresponding author on reasonable request.

## Abbreviations

VHA: Veterans Health Administration
SNFs: Skilled Nursing Facilities
HIR: High-intensity resistance rehabilitation
IRLM: Implementation Research Logic Model
PRISM: Practical Robust Implementation and Sustainability Model
RE-AIM: Reach, Effectiveness, Adoption, Implementation, and Maintenance
ERIC: Expert Recommendation for Implementing Change
PT: Physical therapist
PTA: Physical therapy assistant
OT: Occupational therapist
