# Supplementary material for: Using the implementation research logic model to examine high-intensity resistance rehabilitation implementation in skilled nursing facilities: a mixed methods multi-site case study
Source: Implement Sci Commun. 2025 May 21;6:62. doi: 10.1186/s43058-025-00747-4 (PMC12096742; doi:10.1186/s43058-025-00747-4)
Supplement: Supplementary file 1 — Additional file 1. Description of High-Intensity Resistance Rehabilitation. [file 43058_2025_747_MOESM1_ESM.docx]

**Additional File 1: High-Intensity Resistance Rehabilitation Intervention Description**

High-intensity resistance rehabilitation aims to enhance patient function by applying high-intensity dosing to commonly used physical rehabilitation interventions, such as therapeutic exercise, gait and balance training, and activities of daily living. Dosing targets include an 8-repetition maximum for interventions with countable repetitions. In cases where counting repetitions is not applicable, such as gait tasks within a set time frame, the goal is for patients to successfully complete no more than 80% of the task.

Clinicians are trained to tailor interventions to each patient's specific limitations and goals, allowing the application of high-intensity principles across a wide range of therapeutic activities. They adjust the intensity of these interventions to meet the 8-rep max or 80% target by modifying external load, resistance, prolonging the eccentric phase of movements, or adding environmental barriers or cognitive demands.

In this study, trained physical and occupational therapists, as well as therapy assistants, delivered high-intensity resistance rehabilitation face-to-face in eight rural Veterans Health Administration skilled nursing facilities. They were instructed to apply these principles to all medically appropriate patients throughout their stay, aiming to increase intensity each session. Although fidelity to these principles was not formally assessed, all clinicians demonstrated competency through knowledge and decision-making assessments.

| **Rehabilitation Intervention** | **High-Intensity Resistance Rehabilitation Principles** |
| --- | --- |
| **Therapeutic Exercise** | Clinicians are trained to tailor therapeutic exercises to patients’ impairments and to administer all therapeutic exercises targeting 80% of 1 repetition-max (8RM), for low repetition, high-intensity resistance training. Clinicians are encouraged to progress the intensity when a patient can perform > 8 reps. |
| **Therapeutic Activities:** Activity of Daily Living (ADL) training, Functional Transfer Training | Clinicians are trained to tailor therapeutic activities (e.g., ADL and transfer training) to a patients’ impairments, activity limitations, and rehabilitation goals and to administer activities at an  intensity using an 8RM goal or where patients are successful for no more than 80% of a task. Clinicians are encouraged to progress the intensity when a patient can perform > 8 reps or when they are successful >80% of the activity. |
| **Neuromotor Training** | Clinicians are trained to tailor neuromotor training (e.g., gait and balance training) to a patient’s impairments, activity limitations, and rehabilitation goals and to administer this training at a level of intensity using an 8 RM goal or where patients are successful for no more than 80% of a task. Clinicians are encouraged to progress the intensity when a patient can perform > 8 reps or when they are successful >80% of the activity. |
