# Supplementary material for: Using the implementation research logic model to examine high-intensity resistance rehabilitation implementation in skilled nursing facilities: a mixed methods multi-site case study
Source: Implement Sci Commun. 2025 May 21;6:62. doi: 10.1186/s43058-025-00747-4 (PMC12096742; doi:10.1186/s43058-025-00747-4)
Supplement: Supplementary file 2 — Additional file 2. Description of the Standardized Multicomponent High-Intensity Resistance Rehabilitation Implementation Strategy. [file 43058_2025_747_MOESM2_ESM.docx]

**Additional File 2: Research-led, Multicomponent High-Intensity Resistance Rehabilitation Implementation Strategy**

The following table outlines the distinct components of the 18-week standardized, research-led multicomponent implementation strategy designed to implement high-intensity resistance rehabilitation in skilled nursing facilities. Following an implementation mapping approach, each component was intentionally selected to target a factor (e.g. knowledge, skill, self-efficacy, perspective, resources) that is empirically known or theorized to influence high-intensity resistance rehabilitation implementation outcomes (e.g., adoption and implementation).

| **ERIC Cluster** | **Component Name** | **Definition *(indicating actor, action, temporality, and dose when relevant)*** | **Target *(Justification)*** |
| --- | --- | --- | --- |
| Train and educate stakeholders | Kickoff meeting | Separate, virtual 60-minute meetings with leadership and clinical team prior to initiation of quality improvement program. Meeting objectives were to provide an overview of the quality improvement program including background on the clinical intervention, description of the implementation strategy, overall program targets, and establish clinician and leadership rapport. | Clinician Knowledge |
|  | Online modules | During the initial 12 weeks of the quality improvement program, clinicians engaged with 9 online module presenting didactic content covering high-intensity resistance rehabilitation and completed active learning activities. Module content included a call to action, patient screening and monitoring, principles of muscle overload, dosing and progressing framework, patient engagement strategies, practice efficiency, communication and care continuity, and handling difficult health conditions. Each module, along with its associated learning activities, took between 15 to 90 minutes to complete. Overall, participants dedicated approximately 7 hours over the course of 12 weeks to complete the training | Clinician  Knowledge  Skills  Self-efficacy  Perspectives of intervention |
| Provide interactive assistance | External Implementation Facilitator | A site was assigned a research team member as their External Implementation Facilitator who aided in quality improvement program logistics (e.g., scheduling) and was available for site-specific implementation trouble shooting throughout program duration | Tailor research-led strategies to site-specific needs and/or facilitate introduction of site-led strategies |
|  | Clinical Content Expert | Sites had access to a research team member who served as a Clinical Content Expert who mentored through challenging patient cases throughout program duration | Clinician  Knowledge  Skills  Self-efficacy |
|  | Check ins | Two, 60-minute virtual check-ins occurred during week 4 and week 7 of the 18-week quality improvement program. The External Implementation Facilitator and Clinical Content Expert led the check-ins for each site. Check-ins provided a platform for trouble shooting barriers to high intensity rehabilitation implementation and discussion of patient cases. | Clinician  Knowledge  Skills  Self-efficacy  Perspectives of intervention |
|  | Self-assessments | During the final 6 weeks of the 18-week quality improvement program, clinicians submitted two templated self-assessment and were provided feedback by the External Implementation Facilitator. The self-assessment prompted self-reflection on the execution of a patient encounter where high intensity rehabilitation was implemented and promoted goal setting and action planning. | Clinician  Skills  Self-efficacy |
| Support Clinicians | Tips and tricks | During the final 6 weeks of the program, clinicians received biweekly tips and tricks via email. | Clinician  behavioral nudges and cues |
|  | Job aids | Quick reference pages detailing essential parameters and principles of high intensity resistance rehabilitation. These encompass patient safety, engagement strategies, documentation techniques, and the framework for progressing rehabilitation interventions. | Knowledge  Self-efficacy  Clinician behavioral nudges and cues |
| Change Infrastructure | Provision of necessary equipment | During the first 3 weeks of training, sites received equipment necessary to monitor patient vital signs (e.g. blood pressure cuff, pulse oximeter) and add external resistance to rehabilitation intervention (e.g., weight vests and cuff weights). | Site resources for physical opportunity |

*Description of the implementation strategy follows Proctor et al.’s recommendation for specifying and reporting implementation strategies.*
