# Supplementary material for: Using the implementation research logic model to examine high-intensity resistance rehabilitation implementation in skilled nursing facilities: a mixed methods multi-site case study
Source: Implement Sci Commun. 2025 May 21;6:62. doi: 10.1186/s43058-025-00747-4 (PMC12096742; doi:10.1186/s43058-025-00747-4)
Supplement: Supplementary file 3 — Additional file 3. Questionnaires. [file 43058_2025_747_MOESM3_ESM.docx]

**Additional File 3: Case Study Questionnaires**

| **Demographics** | |
| --- | --- |
| **Age** | *Free text entry* |
| **Gender Identity**  Choose the gender identity or identities with which you identify. | - Male - Female - Non-binary/third gender - Transgender - Cisgender - Agender - Genderqueer - A sex/gender not listed* - Prefer to self-describe* - Prefer not to answer   **if the participant selected a field marked with an asterisk(*), they were asked to provide additional information in free text.* |
| **Race**  Choose the race(s) with which you most closely identify. | - American Indian or Alaska Native - Asian - Black or African-American - Native Hawaiian or Other Pacific Islander - White - Unknown or prefer not to answer |
| **Ethnicity**  Choose the ethnicity with which you most closely identify. | - Hispanic or Latino - Not Hispanic or Latino - Unknown or prefer not to answer |
| **Clinical Role**  What is your clinical role at your facility? | - PT - PTA - OT - COTA - ST - KT - Other*   **if the participant selected a field marked with an asterisk(*), they were asked to provide additional information in free text.* |
| **Clinical Years**  For how many years have you been in clinical practice? | *Free text entry* |
| **Clinical Role Years**  For how many years have you been in your current role? | *Free text entry* |
| **Specialty**  Do you have a specialty certification? | - Yes* - No   **if the participant selected a field marked with an asterisk(*), they were asked to provide additional information in free text.* |
| **Organizational Characteristics** | |
| **Inner Setting** | |
| *This questionnaire consists of scales that measure constructs within the Inner Setting domain of the Consolidated Framework for Implementation Research (CFIR).*  *Please rate your level of agreement with the following statements about your clinical environment.* | |
| *Culture* | |
| People at all levels openly talk about what is and isn't working. | 1. Strongly Disagree 2. Disagree 3. Neutral 4. Agree 5. Strongly Agree |
| Most people in this clinic are willing to change how they do things in response to feedback from others. | 1. Strongly Disagree 2. Disagree 3. Neutral 4. Agree 5. Strongly Agree |
| It is hard to get things to change in our clinic. | 1. Strongly Disagree 2. Disagree 3. Neutral 4. Agree 5. Strongly Agree |
| I can rely on the other people in this clinic to do their jobs well. | 1. Strongly Disagree 2. Disagree 3. Neutral 4. Agree 5. Strongly Agree |
| Most of the people who work in our clinic seem to enjoy their work. | 1. Strongly Disagree 2. Disagree 3. Neutral 4. Agree 5. Strongly Agree |
| Difficult problems are solved through face-to-face discussions. | 1. Strongly Disagree 2. Disagree 3. Neutral 4. Agree 5. Strongly Agree |
| We regularly take time to reflect on how we do things. | 1. Strongly Disagree 2. Disagree 3. Neutral 4. Agree 5. Strongly Agree |
| After trying something new, we take time to think about how it worked. | 1. Strongly Disagree 2. Disagree 3. Neutral 4. Agree 5. Strongly Agree |
| People in this clinic operate as a real team. | 1. Strongly Disagree 2. Disagree 3. Neutral 4. Agree 5. Strongly Agree |
| *Culture Stress* | |
| I am under too many pressures to do my job effectively. | 1. Strongly Disagree 2. Disagree 3. Neutral 4. Agree 5. Strongly Agree |
| Staff members often show signs of stress and strain. | 1. Strongly Disagree 2. Disagree 3. Neutral 4. Agree 5. Strongly Agree |
| The heavy workload here reduces program effectiveness. | 1. Strongly Disagree 2. Disagree 3. Neutral 4. Agree 5. Strongly Agree |
| Staff frustration is common here. | 1. Strongly Disagree 2. Disagree 3. Neutral 4. Agree 5. Strongly Agree |
| *Culture Effort* | |
| People in this clinic always want to perform to the best of their abilities. | 1. Strongly Disagree 2. Disagree 3. Neutral 4. Agree 5. Strongly Agree |
| People are enthusiastic about their work. | 1. Strongly Disagree 2. Disagree 3. Neutral 4. Agree 5. Strongly Agree |
| People in our clinic get by with doing as little as possible. | 1. Strongly Disagree 2. Disagree 3. Neutral 4. Agree 5. Strongly Agree |
| People are prepared to make a special effort to do a good job. | 1. Strongly Disagree 2. Disagree 3. Neutral 4. Agree 5. Strongly Agree |
| People in this clinic do not put more effort into their work than they have to. | 1. Strongly Disagree 2. Disagree 3. Neutral 4. Agree 5. Strongly Agree |
| *Learning Climate* | |
| We regularly take time to consider ways to improve how we do things. | 1. Strongly Disagree 2. Disagree 3. Neutral 4. Agree 5. Strongly Agree |
| People in our clinic actively seek new ways to improve how we do things. | 1. Strongly Disagree 2. Disagree 3. Neutral 4. Agree 5. Strongly Agree |
| This clinic encourages everyone to share ideas. | 1. Strongly Disagree 2. Disagree 3. Neutral 4. Agree 5. Strongly Agree |
| This clinic learns from its mistakes. | 1. Strongly Disagree 2. Disagree 3. Neutral 4. Agree 5. Strongly Agree |
| When we experience a problem in the clinic, we make a serious effort to figure out what's really going on. | 1. Strongly Disagree 2. Disagree 3. Neutral 4. Agree 5. Strongly Agree |
| *Leadership Engagement* |  |
| The clinic leadership makes sure that we have the time and space necessary to discuss changes to improve care. | 1. Strongly Disagree 2. Disagree 3. Neutral 4. Agree 5. Strongly Agree |
| Leadership in this clinic creates an environment where things can be accomplished. | 1. Strongly Disagree 2. Disagree 3. Neutral 4. Agree 5. Strongly Agree |
| Clinic leadership promotes an environment that is an enjoyable place to work. | 1. Strongly Disagree 2. Disagree 3. Neutral 4. Agree 5. Strongly Agree |
| Leadership strongly supports clinic change efforts. | 1. Strongly Disagree 2. Disagree 3. Neutral 4. Agree 5. Strongly Agree |
| **Leadership Communication Satisfaction** | |
| Overall, how satisfied are you with communication between leadership and the rehabilitation team? | 1. Not satisfied 2. Somewhat satisfied 3. Very satisfied 4. I’m not sure/I don’t know |
| **Burnout** | |
| Using your own definition of "burnout," please indicate which statement best describes your situation. | 1. I enjoy my work. I have no symptoms of burnout. 2. Occasionally I am under stress, and I don't have as much energy as I once did, but I don't feel burned out. 3. I am definitely burning out and have one or more symptoms of burnout, such as physical and emotional exhaustion. 4. The symptoms of burnout that I'm experiencing won't go away. I think about frustrations at work a lot. 5. I feel completely burned out and often wonder if I can go on. I am at the point where I may need some changes or may need to seek some sort of help. |
| **Ultrecht Work Engagement Scale-3 (UWES-3)** | |
| *The following 3 statements are about how you feel at work. Please read each statement carefully and decide if you ever feel this way about your job.* | |
| At work, I feel bursting with energy. | 1. Never 2. Almost Never (a few times a year or less) 3. Rarely (once a month or less) 4. Sometimes (a few times a month) 5. Often (once a week) 6. Very Often (a few times a week) 7. Always (every day) |
| I am enthusiastic about my job. | 1. Never 2. Almost Never (a few times a year or less) 3. Rarely (once a month or less) 4. Sometimes (a few times a month) 5. Often (once a week) 6. Very Often (a few times a week) 7. Always (every day) |
| I am immersed in my work. | 1. Never 2. Almost Never (a few times a year or less) 3. Rarely (once a month or less) 4. Sometimes (a few times a month) 5. Often (once a week) 6. Very Often (a few times a week) 7. Always (every day) |
| **Job Satisfaction** | |
| How satisfied are you with your rehabilitation team's ability to restore functional mobility independence in patients at your facility (e.g., gait, ADLs, IADLs, transfers)? | 1. Not Satisfied   5. Completely Satisfied |
| How effective do you believe you are with helping your patients restore functional mobility independence (e.g., gait, ADLs, IADLs, transfers)? | 1. Not Effective   5. Completely Effective |
| *Please rate your level of agreement with the following statements:* | |
| Please rate your level of agreement with the following statement: I find my current work personally rewarding. | 1. Completely Disagree   5. Completely Agree |
| Please rate your level of agreement with the following statement: Overall, I am satisfied in my current practice. | 1. Completely Disagree   5. Completely Agree |
| **Provider Perceptions of Team Effectiveness (Provider PTE)** | |
| *These next questions will ask you about the culture and climate at your SNF.*  *Please rate your level of agreement with the following statements.* | |
| I trust other members of the healthcare team. | 1. Strongly Disagree 2. Disagree 3. Disagree Somewhat 4. Agree Somewhat 5. Agree 6. Strongly Agree |
| My healthcare team is effective in providing patient care. | 1. Strongly Disagree 2. Disagree 3. Disagree Somewhat 4. Agree Somewhat 5. Agree 6. Strongly Agree |
| Team members share relevant information to inform patient care decisions. | 1. Strongly Disagree 2. Disagree 3. Disagree Somewhat 4. Agree Somewhat 5. Agree 6. Strongly Agree |
| My ideas, information or observations are used to solve patient care issues. | 1. Strongly Disagree 2. Disagree 3. Disagree Somewhat 4. Agree Somewhat 5. Agree 6. Strongly Agree |
| Healthcare team members know the goals of patients' plans of care. | 1. Strongly Disagree 2. Disagree 3. Disagree Somewhat 4. Agree Somewhat 5. Agree 6. Strongly Agree |
| The patient's health record is updated as required. | 1. Strongly Disagree 2. Disagree 3. Disagree Somewhat 4. Agree Somewhat 5. Agree 6. Strongly Agree |
| The flow of information between team members and patients and families is constrained. | 1. Strongly Disagree 2. Disagree 3. Disagree Somewhat 4. Agree Somewhat 5. Agree 6. Strongly Agree |
| The healthcare team adjusts treatments according to changes in the patient's condition. | 1. Strongly Disagree 2. Disagree 3. Disagree Somewhat 4. Agree Somewhat 5. Agree 6. Strongly Agree |
| The care provided by the healthcare team is well organized. | 1. Strongly Disagree 2. Disagree 3. Disagree Somewhat 4. Agree Somewhat 5. Agree 6. Strongly Agree |
| Team members work together to solve patient care issues. | 1. Strongly Disagree 2. Disagree 3. Disagree Somewhat 4. Agree Somewhat 5. Agree 6. Strongly Agree |
| Disagreements among team members are dealt with fairly by team members. | 1. Strongly Disagree 2. Disagree 3. Disagree Somewhat 4. Agree Somewhat 5. Agree 6. Strongly Agree |
| Differences of opinion among team members are respected. | 1. Strongly Disagree 2. Disagree 3. Disagree Somewhat 4. Agree Somewhat 5. Agree 6. Strongly Agree |
| I have a role to play in the team. | 1. Strongly Disagree 2. Disagree 3. Disagree Somewhat 4. Agree Somewhat 5. Agree 6. Strongly Agree |
| My contributions are valued by my healthcare team. | 1. Strongly Disagree 2. Disagree 3. Disagree Somewhat 4. Agree Somewhat 5. Agree 6. Strongly Agree |
| Working with families to solve patient care issues is not part of the team's mandate. | 1. Strongly Disagree 2. Disagree 3. Disagree Somewhat 4. Agree Somewhat 5. Agree 6. Strongly Agree |
| **Team Communication** | |
| How does your team communicate about patient/handle patient handoffs?  *(Select all that apply)* | - Formal team huddles - Informal discussion - Written documentation/patient notes - Other (please indicate)* - Prefer not to answer   **if the participant selected a field marked with an asterisk(*), they were asked to provide additional information in free text.* |
| How does your team communicate about a patient's status?  *(Select all that apply)* | - Formal team huddles - Informal discussion - Written documentation/patient notes - Other (please indicate)* - Prefer not to answer   **if the participant selected a field marked with an asterisk(*), they were asked to provide additional information in free text.* |
| How frequently does your team hold formal team huddles/meetings? | - Daily - Weekly - 2-3 times a week - 3-4 times a week - Twice a month - Other (please indicate)*   **if the participant selected a field marked with an asterisk(*), they were asked to provide additional information in free text.* |
| Overall, how satisfied are you with your rehabilitation team’s communication frequency? | 1. Not satisfied 2. Somewhat satisfied 3. Very satisfied 4. I’m not sure/I don’t know |
| Overall, how satisfied are you with your rehabilitation team’s communication quality? | 1. Not satisfied 2. Somewhat satisfied 3. Very satisfied 4. I’m not sure/I don’t know |
| **Clinician Characteristics** | |
| **Self-Efficacy** | |
| *Please indicate your level of confidence with each of the following before and after participating in this training experience.*    *When answering these items, please think about a typical patient that you might encounter in your practice (as opposed to a unique or rare patient) unless otherwise indicated. Also, consider your answers in the context of your day-to-day workflow and work environment.*    *For the below:*    *1 = no confidence*  *5 = total confidence*  *N/A for not applicable* | |
| *Screening and Monitoring* | |
| *Correctly identifying patients who are appropriate for high-intensity rehabilitation.* | |
| Before training: | 1. No Confidence   5. Total Confidence   N/A |
| After training: | 1. No Confidence   5. Total Confidence   N/A |
| *Conducting ongoing monitoring (e.g., of vital signs and exertion levels) to determine safe continued participation in high-intensity rehabilitation.* | |
| Before training: | 1. No Confidence   5. Total Confidence   N/A |
| After training: | 1. No Confidence   5. Total Confidence   N/A |
| *High-Intensity Dosing and Progression* | |
| *Consistently applying individualized, high-intensity dosing to therapeutic exercises.* | |
| Before training: | 1. No Confidence   5. Total Confidence   N/A |
| After training: | 1. No Confidence   5. Total Confidence   N/A |
| *Consistently applying individualized, high-intensity dosing to therapeutic activities.* | |
| Before training: | 1. No Confidence   5. Total Confidence   N/A |
| After training: | 1. No Confidence   5. Total Confidence   N/A |
| *Consistently applying individualized, high-intensity dosing to neuromotor activities (e.g., gait and balance training).* | |
| Before training: | 1. No Confidence   5. Total Confidence   N/A |
| After training: | 1. No Confidence   5. Total Confidence   N/A |
| *Progressing high-intensity interventions within a session to achieve failure with each intervention.* | |
| Before training: | 1. No Confidence   5. Total Confidence   N/A |
| After training: | 1. No Confidence   5. Total Confidence   N/A |
| *Progressing high-intensity interventions between sessions to achieve failure with each intervention.* | |
| Before training: | 1. No Confidence   5. Total Confidence   N/A |
| After training: | 1. No Confidence   5. Total Confidence   N/A |
| *Communication/Ensuring Continuity of Care* | |
| *Readily and clearly communicating (verbally and in writing) a patient's eligibility for and response to high-intensity rehabilitation with the rehabilitation team.* | |
| Before training: | 1. No Confidence   5. Total Confidence   N/A |
| After training: | 1. No Confidence   5. Total Confidence   N/A |
| *Readily and clearly communicating (verbally and in writing) recommendations for progressing a patient using high-intensity rehabilitation with the rehabilitation team.* | |
| Before training: | 1. No Confidence   5. Total Confidence   N/A |
| After training: | 1. No Confidence   5. Total Confidence   N/A |
| *Coping, Adapting, Addressing Barriers, Problem Solving* | |
| *Adapting to and intervening appropriately to allow high-intensity rehabilitation to continue following a change in patient status or subjective report (e.g., during episodes of shortness of breath, pain, fear/anxiety with movement).* | |
| Before training: | 1. No Confidence   5. Total Confidence   N/A |
| After training: | 1. No Confidence   5. Total Confidence   N/A |
| *Utilizing patient education and motivational strategies to maximize patient engagement in high-intensity rehabilitation.* | |
| Before training: | 1. No Confidence   5. Total Confidence   N/A |
| After training: | 1. No Confidence   5. Total Confidence   N/A |
| *Adapting your approach to apply high-intensity rehabilitation to group and concurrent rehabilitation sessions.* | |
| Before training: | 1. No Confidence   5. Total Confidence   N/A |
| After training: | 1. No Confidence   5. Total Confidence   N/A |
| *Maintaining effective time management as you integrate high-intensity rehabilitation into each patient session throughout your workday.* | |
| Before training: | 1. No Confidence   5. Total Confidence   N/A |
| After training: | 1. No Confidence   5. Total Confidence   N/A |
| **Evidence-based Practice Attitudes Scale (EBPAS)** | |
| *Please rate your level of agreement with the following statements:* | |
| I like to use new types of therapy/interventions to help my clients. | 1. Not at all 2. To a slight extent 3. To a moderate extent 4. To a great extent 5. To a very great extent |
| I am willing to try new types of therapy/interventions even if I have to follow a treatment manual. | 1. Not at all 2. To a slight extent 3. To a moderate extent 4. To a great extent 5. To a very great extent |
| I know better than academic researchers how to care for my clients. | 1. Not at all 2. To a slight extent 3. To a moderate extent 4. To a great extent 5. To a very great extent |
| I am willing to use new and different types of therapy/interventions developed by researchers. | 1. Not at all 2. To a slight extent 3. To a moderate extent 4. To a great extent 5. To a very great extent |
| Research based treatments/interventions are not clinically useful. | 1. Not at all 2. To a slight extent 3. To a moderate extent 4. To a great extent 5. To a very great extent |
| Clinical experience is more important than using manualized therapy/interventions. | 1. Not at all 2. To a slight extent 3. To a moderate extent 4. To a great extent 5. To a very great extent |
| I would not use manualized therapy/interventions. | 1. Not at all 2. To a slight extent 3. To a moderate extent 4. To a great extent 5. To a very great extent |
| I would try a new therapy/intervention even if it were very different from what I am used to doing. | 1. Not at all 2. To a slight extent 3. To a moderate extent 4. To a great extent 5. To a very great extent |
| *For the following, if you received training in a therapy or intervention that was new to you, how likely would you be to adopt it if...* | |
| …it was intuitively appealing? | 1. Not at all 2. To a slight extent 3. To a moderate extent 4. To a great extent 5. To a very great extent |
| …it "made sense" to you? | 1. Not at all 2. To a slight extent 3. To a moderate extent 4. To a great extent 5. To a very great extent |
| …it was required by your supervisor? | 1. Not at all 2. To a slight extent 3. To a moderate extent 4. To a great extent 5. To a very great extent |
| …it was required by your agency? | 1. Not at all 2. To a slight extent 3. To a moderate extent 4. To a great extent 5. To a very great extent |
| …it was required by your state? | 1. Not at all 2. To a slight extent 3. To a moderate extent 4. To a great extent 5. To a very great extent |
| …it was being used by colleagues who were happy with it? | 1. Not at all 2. To a slight extent 3. To a moderate extent 4. To a great extent 5. To a very great extent |
| …you felt you had enough training to use it correctly? | 1. Not at all 2. To a slight extent 3. To a moderate extent 4. To a great extent 5. To a very great extent |
| **Implementation and Sustainability Infrastructure** | |
| **Implementation Climate** | |
| Clinic staff are expected to help the High-Intensity Rehabilitation Program meet its goal (i.e., improve patient outcomes through screening for patient appropriateness for high-intensity rehabilitation and applying high-intensity dosing to rehabilitation interventions). | 1. Strongly Disagree 2. Disagree 3. Neutral 4. Agree 5. Strongly Agree |
| Clinic staff get the support they need to implement high-intensity rehabilitation. | 1. Strongly Disagree 2. Disagree 3. Neutral 4. Agree 5. Strongly Agree |
| Clinic staff get recognition for implementing high-intensity rehabilitation to improve patient outcomes. | 1. Strongly Disagree 2. Disagree 3. Neutral 4. Agree 5. Strongly Agree |
| Providing care in line with high-intensity rehabilitation to improve patient outcomes is a top priority of the clinic. | 1. Strongly Disagree 2. Disagree 3. Neutral 4. Agree 5. Strongly Agree |
| **Change Capacity** | |
| *On a scale of 1-5, with 1 being totally disagree and 5 being totally agree, please rate your level of agreement with the following statements.* | |
| We/I have the resources required to implement these changes to rehabilitation approaches in this facility. | 1. Totally Disagree   5. Totally Agree |
| We/I have the time necessary to implement these changes to rehabilitation approaches in this facility. | 1. Totally Disagree   5. Totally Agree |
| We/I have the necessary support from leadership to implement these changes to rehabilitation approaches. | 1. Totally Disagree   5. Totally Agree |
| We/I have the necessary communication channels in place to implement these changes to rehabilitation approaches. | 1. Totally Disagree   5. Totally Agree |
| High-intensity rehabilitation is necessary to improve patient outcomes and quality of care provided in this facility. | 1. Totally Disagree   5. Totally Agree |
| **Clinician Perspective** | |
| **Perceived Characteristics of Intervention (PCIS)** | |
| *The following questions assess your opinion of high-intensity rehabilitation.* | |
| High-intensity rehabilitation is more effective than other therapies that I have used. | 1. Strongly Disagree 2. Disagree 3. Neither Agree Nor Disagree 4. Agree 5. Strongly Agree |
| High-intensity rehabilitation is more convenient than other therapies I have used. | 1. Strongly Disagree 2. Disagree 3. Neither Agree Nor Disagree 4. Agree 5. Strongly Agree |
| Using high-intensity rehabilitation fits with the way I like to work. | 1. Strongly Disagree 2. Disagree 3. Neither Agree Nor Disagree 4. Agree 5. Strongly Agree |
| High-intensity rehabilitation is aligned with my clinical judgment. | 1. Strongly Disagree 2. Disagree 3. Neither Agree Nor Disagree 4. Agree 5. Strongly Agree |
| High-intensity rehabilitation is clear and understandable. | 1. Strongly Disagree 2. Disagree 3. Neither Agree Nor Disagree 4. Agree 5. Strongly Agree |
| High-intensity rehabilitation is easy to perform. | 1. Strongly Disagree 2. Disagree 3. Neither Agree Nor Disagree 4. Agree 5. Strongly Agree |
| High-intensity rehabilitation can be tested out with patients without disrupting their overall therapy. | 1. Strongly Disagree 2. Disagree 3. Neither Agree Nor Disagree 4. Agree 5. Strongly Agree |
| It is easy to tell whether patients are benefitting from high-intensity rehabilitation. | 1. Strongly Disagree 2. Disagree 3. Neither Agree Nor Disagree 4. Agree 5. Strongly Agree |
| High-intensity rehabilitation can be adapted to my treatment setting. | 1. Strongly Disagree 2. Disagree 3. Neither Agree Nor Disagree 4. Agree 5. Strongly Agree |
| High-intensity rehabilitation can be adapted to meet the needs of my patients. | 1. Strongly Disagree 2. Disagree 3. Neither Agree Nor Disagree 4. Agree 5. Strongly Agree |
| It is easy to try out high-intensity rehabilitation and see how it performs. | 1. Strongly Disagree 2. Disagree 3. Neither Agree Nor Disagree 4. Agree 5. Strongly Agree |
| Using high-intensity rehabilitation includes a risk of worsening patients' symptoms. | 1. Strongly Disagree 2. Disagree 3. Neither Agree Nor Disagree 4. Agree 5. Strongly Agree |
| Using high-intensity rehabilitation includes a risk of doing more harm than good. | 1. Strongly Disagree 2. Disagree 3. Neither Agree Nor Disagree 4. Agree 5. Strongly Agree |
| Using high-intensity rehabilitation improves the quality of the work that I do. | 1. Strongly Disagree 2. Disagree 3. Neither Agree Nor Disagree 4. Agree 5. Strongly Agree |
| High-intensity rehabilitation produces improvements that I can actually see in my patients. | 1. Strongly Disagree 2. Disagree 3. Neither Agree Nor Disagree 4. Agree 5. Strongly Agree |
| Using high-intensity rehabilitation makes it easier to do my job. | 1. Strongly Disagree 2. Disagree 3. Neither Agree Nor Disagree 4. Agree 5. Strongly Agree |
| The knowledge required to learn high-intensity rehabilitation can be effectively taught. | 1. Strongly Disagree 2. Disagree 3. Neither Agree Nor Disagree 4. Agree 5. Strongly Agree |
| The skills required to implement high-intensity rehabilitation can be effectively taught. | 1. Strongly Disagree 2. Disagree 3. Neither Agree Nor Disagree 4. Agree 5. Strongly Agree |
| The high-intensity rehabilitation course and supporting materials are helpful. | 1. Strongly Disagree 2. Disagree 3. Neither Agree Nor Disagree 4. Agree 5. Strongly Agree |
| **Site Implementation** | |
| **Provider Report of Sustainment Scale (PRESS)** | |
| *The following questions ask about high-intensity rehabilitation in your facility. Please indicate the extent to which you agree with the following items.*    *(0=Not at all and 4 = To a very great extent)* | |
| Staff use high-intensity rehabilitation as much as possible, when appropriate. | 1. Not at all   5. To a very great extent |
| Staff continue to use high-intensity rehabilitation throughout challenging circumstances. | 1. Not at all   5. To a very great extent |
| High-intensity rehabilitation is a routine part of our practice. | 1. Not at all   5. To a very great extent |
