# Supplementary material for: Using the implementation research logic model to examine high-intensity resistance rehabilitation implementation in skilled nursing facilities: a mixed methods multi-site case study
Source: Implement Sci Commun. 2025 May 21;6:62. doi: 10.1186/s43058-025-00747-4 (PMC12096742; doi:10.1186/s43058-025-00747-4)
Supplement: Supplementary file 4 — Additional file 4. Qualitatively Identified PRISM Constructs and Factors by Site Implementation Extent. [file 43058_2025_747_MOESM4_ESM.docx]

**Additional File 4.** Qualitatively Identified PRISM Constructs and Contextual Factors by Site Implementation Extent.

| **Construct** | **Factors** | **Description** | **Quartile differences** |
| --- | --- | --- | --- |
| External Environment | COVID | Lighter patient caseload (F)    Caseload with increased medical complexity and poor mental health; staffing fluctuations; scheduling challenges; environmental instability (B) | Lower implementing sites reported COVID as a one of their strongest barriers. |
|  | Critical difference between VA and private sector | VA provided autonomy, access to hospital medical records, care model, and patient familiarity with physical demands (F) | No pattern detected |
|  | Stigma | Clinician assumptions of what patients can tolerate based on their age (B) | No pattern identified |
|  | Patient evolution | Clinicians see trends of sicker patients desiring to discharge home (F)    Clinicians and leaders indicate trends of “sicker” “more complex” patients being admitted to SNF (B) | Higher implementing sites leveraged a shared trend as a facilitator |
| Organizational characteristics | Operations | Scheduling autonomy and flexibility with optimized care continuity (F)    Treatment duration of 30 minutes or less (B)  Not having a caseload on CLC or frequent, drastic fluctuations of caseload (B) | Higher implementing sites had scheduling autonomy and flexibility, a schedule/care model that optimized continuity, and a more stable caseload and staffing levels |
|  | Leadership | “pro rehab” mindset of upper management; mutual respect between clinicians and leadership through acknowledgement and opportunities to express self (F)    Perceptions of communication, presence, awareness, and acknowledgement that does not meet needs (B) | Higher implementing sites shared more general positive experiences with leadership; lower implementing sies shared less positive experiences with leadership |
|  | Team culture | Collaborative rehabilitation team attributed to longevity, tenure, sharing of physical spaces, frequent touchpoints (F)    Relationship, collaboration, and accessibility to interdisciplinary team (F) | All teams presented theme as facilitator |
| Clinician Characteristics | Demeanor | Enthusiasm, love for role, work ethic (F) | Leadership from higher implementing sites reported more frequently |
|  | **Inertia of existing practice** | Tendency to fall back on previous “habits” of lower intensity rehabilitation (B)    Prior practice patterns similar to high-intensity resistance rehabilitation (F) | Higher implementing site had existing practices more parallel to high-intensity resistance rehabilitation |
|  | Openness | Leaders indicate team is open to change (F)    Leaders highlight general resistance to change (B) | Leadership at lower implementing site reflected on teams lack of openness to change |
| Clinician Perspective | **Compatibility** | Considered high-intensity resistance rehabilitation as compatible with SNF (F) but not with most patients or clinical responsibilities (B) | Higher implementing sites indicated as a barrier less frequently |
|  | Relative advantage/value | Clinicians agreed high-intensity resistance rehabilitation can help patients achieve functional outcomes faster (F) | All sites presented theme as facilitator |
|  | **Complexity** | Simple conceptually but not easy to implement. Remembering to implement, session planning, knowing where to start or how to progress patients, and increased time and cognitive demands (B) | Lower implementing sites indicated less challenges with complexity |
|  | Adaptability | Ability to apply high-intensity resistance rehabilitation as framework to apply across diverse contexts (F) | Lowest implementing site will more examples of using principles as protocol vs framework |
| Patient characteristics | **Mindset** | Patients with opportunity to and motivation of returning home more willing to participate (F)    Patients without a goal of returning home, patients with prior experience of and preference for low-intensity rehabilitation, and patients with plan of care competing priorities less willing to participate (B) | Higher implementing sites indicate more patients embracing high-intensity resistance rehabilitation |
|  | Cognition | Cognitive impairments (B) | All sites highlight as barrier; lower implementing sites indicate most frequently |
| Infrastructure | **Leadership support** | Leadership acknowledgement, championing, and actively facilitating implementation (F)    Lack of leadership presence, awareness, acknowledgement, and prioritization of high-intensity resistance rehabilitation implementation (B) | Higher implementing sites presented stronger examples of leadership support; lower implementing sites requested greater leadership support |
|  | Resources | Lack of resistance equipment and gym space (B) | All sites indicate theme as barrier |
|  | Sharing best practices | Teams leveraged existing social networks and communication channels to discuss high-intensity resistance rehabilitation implementation (F) | All sites indicate existing, strong communication channels used to discuss high-intensity resistance rehabilitation |
|  | Dedicated team | Team goes/went above and beyond generally and for this initiative (F) | Leadership from higher implementing sites pointed to team dedication as strongest influence |

(F) indicates implementation facilitator, (B) indicates implementation barrier. Bolded themes indicate those perceived as most influential and/or targeted by site-initiated implementation strategies.
