# Supplementary material for: Using the implementation research logic model to examine high-intensity resistance rehabilitation implementation in skilled nursing facilities: a mixed methods multi-site case study
Source: Implement Sci Commun. 2025 May 21;6:62. doi: 10.1186/s43058-025-00747-4 (PMC12096742; doi:10.1186/s43058-025-00747-4)
Supplement: Supplementary file 5 — Additional file 5. Site-Initiated Implementation Strategy Description and Mechanisms. [file 43058_2025_747_MOESM5_ESM.docx]

**Additional File 5:** Site-Initiated Implementation Strategy Descriptions and Mechanism

| **Strategy** | **Description** | **Mechanism***  (if identified) | **Example** |
| --- | --- | --- | --- |
| Evaluative and iterative strategies | Early full team engagement | - Clinician perspective (buy-in) | *It sounds like we just needed to get the -- the PTAs involved sooner. And, um, I don't think anybody ever didn't recognize their importance in the process. But, um, I -- I think it's -- was maybe harder to get buy ins since they weren't involved from the beginning and involved in the decision to do it. And, um, I think, you know, maybe better buy in from them from the start would have been helpful* (Upper Management 7) |
|  | Patient outcomes collection | - Positive patient environment - Early positive reinforcement |  |
| Infrastructure change | Protected time | - Decreased cognitive load with treatment planning and execution - Enhanced adaptation capacity and confidence - Early positive reinforcement | *I think it would be nice, I guess, as part of our documentation if we had a specific prompt maybe in our documentation template that kind of triggered -- I don’t know whether it’s cosigning them, or whether it’s this is the part we’re focused on, and this is what we’re doing, and here’s what to do next. Because I think sometimes prompting yourself to document the next steps to this is how I want to challenge the patient next, might be a good -- I mean, to me that’s a good way to not only keep yourself accountable for doing it, but also getting everybody else on board. This is the direction we’re headed, and making sure that we’re all doing that. (PT 7)* |
|  | Documentation adjustments: *assistants sign off on treatment notes, documentation sheets for temporary staff, specificity of intervention description and plan.* | - Decreased cognitive load with treatment planning and execution - Care continuity - Salience |  |
| Engage consumers | Patient education and engagement | - Positive patient environment | *with engagement…if I can say, you know, we're going to do this task in this manner because we're working towards blah, blah, blah, um, they're more willing to participate. KT 6* |
|  | Group therapy: *a single therapist provides rehabilitation to a group of patients simultaneously* | - Positive patient environment (*social normalization, positive perspectives, enthusiasm)* |  |
|  | Co-treat: >1 *clinician treats a patient side by side* | - Positive patient environment (*patient confidence and trust)* |  |
| Stakeholder interrelationships | Frequent clinician high-intensity resistance rehabilitation communication | - Salience - Clinician perspective - Clinician adaptation capacity and confidence | *PTA 5.1 and I would say, okay, we got one. So let's share. (PT 5)* |
|  | Increase specificity of verbal handoffs | - Decrease cognitive effort required for treatment planning and execution - Care continuity |  |
|  | Patient sharing: *(clinicians from same discipline trade treatment sessions)* or co-treat: *clinician treats a patient side by side* | - Clinician adaptation capacity and confidence |  |
|  | Communicate high-intensity resistance rehabilitation interdisciplinary team | - Positive clinician environment - Positive patient environment |  |
| Interactive assistance and support clinicians | Informal champion or internal facilitator: *leader or clinician spontaneously fulfills role* | - Enhanced adaptation capacity and confidence - Clinician perspective - Early positive reinforcement - Salience | *And I don't think that we -- like, PT7.1 and I need much, um, more cheerleading. I think -- I mean, I think we're pretty well sold on it. We just might have to be the cheerleaders for the rest of the team to, like, continue with it. Um, so whether -- and I think implementing all the strategies that PT 7.1 mentioned. I think that will be a good -- a good way to just hold everybody accountable, like she said. And, um -- and then, yeah. We might get annoying with it, but that's okay. Like, hey, you could probably throw the vest on this person (OT 7)*    *I would have liked to have done some co-treats with the PTs. Um, just to see how they're inter- -- implementing the interventions and what are possible modifications we could do to them. Um, but because of that, I wasn't able to do co-treats with them. Everyone was doing one-on-ones. Um, so I think I would have liked to have done that (PT 8)* |

**Mechanism refers to how the implementation strategy influenced implementation outcomes*
